# Evaluation of maternal serum fibroblast growth factor-23 levels in fetal growth restriction and gestational hypertensive disease

**DOI:** 10.1590/1806-9282.20231496

**Published:** 2024-07-19

**Authors:** Erdener Karacan, Ümran Kılınçdemir Turgut, Halil İbrahim Büyükbayram, Mehmet Güney

**Affiliations:** 1 Süleyman Demirel University, Faculty of Medicine, Department of Obstetrics and Gynecology – Isparta, Turkey.; 2 Adana City Training and Research Hospital, Department of Obstetrics and Gynaecology – Adana, Turkey.; 3 Süleyman Demirel University, Faculty of Medicine, Department of Biochemistry – Isparta, Turkey.; 4 Dokuz Eylul University, Department of Obstetrics and Gynaecology – İzmir, Turkey.

**Keywords:** Gestational hypertension, Fibroblast Growth Factor-23, Pregnancy, Fetal growth restriction

## Abstract

**OBJECTIVE:**

The objective of this study was to determine serum fibroblast growth factor-23 levels in preeclampsia, eclampsia, gestational hypertension, and the presence of fetal growth restriction subgroups.

**METHODS:**

A total of 55 pregnant women with planned cesarean section were included in this cross-sectional study. They were divided into two groups, namely, control (25) and gestational hypertensive disease (30). The gestational hypertensive disease group was evaluated by dividing it into three subgroups (preeclampsia, eclampsia, and gestational hypertension) according to the clinical and laboratory findings of the disease and two subgroups (presence of fetal growth restriction and absence of fetal growth restriction) according to the birth weight percentile. Demographic parameters, obstetric history, physical examination findings, and laboratory values were evaluated.

**RESULTS:**

Demographic parameters and obstetric history were similar between the two groups, while gestational week of delivery was lower in the gestational hypertensive disease group (p=0.002). Laboratory parameters and serum fibroblast growth factor-23 (pg/mL) values were similar between the two groups. In the subgroup analysis for gestational hypertension, preeclampsia, and eclampsia, there was no statistically significant difference in serum fibroblast growth factor-23 levels between gestational hypertension, preeclampsia, eclampsia, and control groups. In the subgroup analysis based on the presence of fetal growth restriction, serum fibroblast growth factor-23 levels were similar to the control group in the gestational hypertensive disease absence of fetal growth restriction, while serum fibroblast growth factor-23 levels and serum calcium levels were statistically significantly lower in the gestational hypertensive disease with the presence of fetal growth restriction (p=0.044 and p<0.001, respectively).

**Conclusion:**

Serum fibroblast growth factor-23 levels are similar between pregnancies complicated with gestational hypertensive disease and normotensive pregnancies. However, serum fibroblast growth factor-23 levels were found to be lower in pregnancies complicated with gestational hypertensive disease with the presence of fetal growth restriction.

## INTRODUCTION

Gestational hypertensive disease (GHD), with or without proteinuria, is the onset of high blood pressure (systolic ≥ 140 mmHg; diastolic ≥ 90 mmHg) after 20 weeks of gestation^1^, and it is one of the main causes of maternal mortality and morbidity. GHD is closely associated with poor perinatal outcomes, including fetal growth retardation and low birth weight (BW)^2^. Gestational hypertension (GHT) and preeclampsia/eclampsia are in the group of GHDs^3^. GHD/preeclampsia has been considered to have different pathophysiological pathways because of its different clinical courses and different fetal and maternal outcomes, according to clinical findings^4^.

Recently, to understand the pathophysiology of GHD, the placental expression of the promoter region of Klotho in preeclamptic pregnant women was studied^5^. Three Klotho-related genes have been identified: α-Klotho, β-Klotho, and γ-Klotho^6^. Studies have shown that, in preeclamptic pregnancies, there is genotyping polymorphism and decreased expression of the Klotho gene^7^. In addition, maternal plasma α-Klotho elevation in preeclamptic pregnant women was associated with less placental villous maturation. Therefore, changes in the a-Klotho system are thought to be involved in the etiology of preeclampsia^8^.

Fibroblast growth factor-23 (FGF23) is a member of the hormonal FGF subfamily that complexes with Klotho co-receptors^9^. FGF23 is likely to act on the FGFR1c receptor and, together with α-Klotho, has a 20-fold greater affinity for the FGFR receptor^10^.

An increase in serum FGF23 levels results in hypophosphatemia, a decrease in 1–25 (OH)_2_ D levels, and a decrease in bone mineral density, while a decrease results in hyperphosphatemia and an increase in 1–25 (OH)_2_ D levels and soft tissue calcifications^11^. Part of the known physiological function of FGF23 in regulating mineral metabolism can also be explained by the effects of this hormone on the kidney. FGF23 is a regulator of the sodium chloride channel in the distal renal tubules, is known to cause renal sodium retention, plasma expansion, and increases in systolic and diastolic blood pressure^12^.

The aim of this study was to determine the levels of serum FGF23 in pregnancies complicated with GHD and investigate FGF23 levels in preeclampsia, eclampsia, GHT, and the presence of fetal growth restriction (FGR) subgroups.

## METHODS

The study design was approved by the local ethics committee (23.03.2021 8/152), and informed consent was obtained from all participants. Pregnant women for whom a cesarean delivery was planned were included in the study. The control group comprised pregnant women who had had a previous cesarean delivery, were normotensive, and had no maternal or fetal risks during their pregnancy.

**Figure 1:**
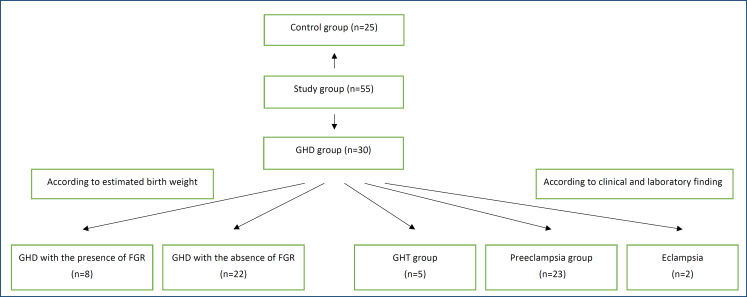
Study plan and flowchart. GHT: gestational hypertension; GHD: gestational hypertensive disease; FGR: fetal growth restriction.

The GHD group included pregnant women who were known to be normotensive before the 24th week of pregnancy and who were found to have systolic/diastolic blood pressures higher than 140/90 mmHg in two measurements measured at 4-h intervals. The GHD group was first divided into three subgroups according to GHD symptoms and laboratory findings as follows: (1) the preeclampsia subgroup had hypertension and proteinuria over 300 mg/dL in 24-h urine; (2) the GHT subgroup had hypertension without proteinuria; and (3) the eclampsia subgroup had convulsions in addition to the preeclampsia group. The GHD group was also divided into two subgroups according to BW. In the first subgroup, patients showed an absence of FGR (BW>10%), while in the second subgroup, patients showed a presence of FGR (BW<10%) (Figure 1).

The exclusion criteria included the following: multiple pregnancies, genetic or structural fetal anomaly, diabetes mellitus, gestational diabetes mellitus (GDM), chronic hypertensive disease, chronic kidney disease, autoimmune and rheumatic disease, the presence of thyroid disease (hypothyroidism or hyperthyroidism), delivery before the 32nd gestational week, smoking and alcohol use, and vaginal delivery.

### Demographic parameters and obstetric/physical examination findings

The following variables were evaluated: demographic parameters and obstetric/physical examination findings, age, gravity, parity, abortion, number of fetuses, gestational week, estimated fetal weight, BW, amniotic fluid amount, and arterial blood pressure (systolic/diastolic).

### Laboratory parameters and biochemical measurements

Maternal hemoglobin (Hgb) (g/dl) measurements were evaluated before delivery and at the sixth hour after delivery. Prenatal maternal venous blood samples were taken, and the blood serum was separated and analyzed within 12 h. Maternal serum calcium (Ca) (mg/dl), serum phosphate (Pi) (mg/dL), corrected Ca (mg/dl), and serum FGF23 (pg/mL) were measured. Serum FGF23 levels were studied following the ELISA kit procedure (Cat. No. E0059Hu, Lot No. 202109012, Shanghai Korain BT Laboratory, Jiaxing, China). The intra-assay CV (intra-measurement coefficient of variation) was <8%, while the inter-assay CV (inter-measurement coefficient of variation) was <10%.

### Statistical analysis

Statistical analysis of the study data was performed using the IBM SPSS Statistics software (IBM Corporation, Armonk, NY, USA). Whether the data showed normal distribution or not was evaluated using the Shapiro-Wilk test. The data were observed to have a normal distribution, and Levene's test of homogeneity was used to evaluate whether the variances provided a homogeneous distribution. Then, comparisons between groups were made using parametric tests. The Student's t-test and one-way ANOVA were used to compare the analyzed findings of the groups. The Kruskal-Wallis and the Mann-Whitney U tests were used for data with inhomogeneous variances and small sample size. Statistical significance was considered to be p<0.05 at a 95% confidence interval.

## RESULTS

A total of 55 pregnant women who met the inclusion and exclusion criteria of the study were included. Notably, 25 pregnant women were included in the control group and 30 pregnant women were included in the GHD group, which was further sub-classified according to laboratory parameters and physical examination findings: 23 pregnant women were in preeclampsia subgroup, 5 pregnant women were in the GHT subgroup, and 2 pregnant women were in the eclampsia subgroup. Moreover, the GHD group was further subclassified according to fetal birth measurement: FGR was present in 8 participants and absent in 22 participants.

Demographic parameters, obstetric examinations, and neonatal outcomes were similar in the control and GHD groups, except for the gestational week at delivery, which was statistically significantly earlier in the GHD group (p=0.002) (Table 1). Maternal blood pressure and laboratory parameters of the control and GHD groups were compared, and systolic and diastolic blood pressure was significantly higher in the GHD group (p<0.001). Laboratory parameters, including serum albumin levels, serum calcium levels, albumin-corrected calcium levels, and serum phosphate levels, were similar in the two groups (Table 1).

**Table 1:** Comparison of the gestational hypertensive disease and the control groups in terms of demographic parameters and laboratory values.

	Control (25)	GHD (30)	p-value
	Mean±standard deviation		
Age (years)	30.72±6.33	29.97±6.36	NS
Gravity	2.60±1.44	2.57±1.81	NS
Parity	0.80±0.76	0.93±1.08	NS
Abortus	0.84±0.98	0.77±1.40	NS
Birth weight (g)	3126.60±371.24	2903.00±775.62	0.379
Birth weight percentile (%)	45.08±28.48	46.33±34.33	0.735
Gestational week at delivery	38.00±1.53	36.50±2.03	0.002*
Preop Hgb (g/dl)	12.62±1.11	12.1±0.95	0.082
Postop Hgb (g/dL)	11.12±1.26	10.56±1.43	0.137
Serum Ca (mg/dL)	8.80±0.48	8.47±0.68	0.052
Corrected Ca (mg/dL)	9.33±0.38	9.18±0.55	0.253
Serum Pi (mg/dL)	3.54±0.53	3.78±0.91	0.577
Systolic (mmHg)	104.40±12.61	153.33±16.47	<0.001*
Diastolic (mmHg)	67.20±7.91	99.00±11.55	<0.001*
FGF23 (pg/mL)	285.09±218.18	248.39±305.45	0.265

GHD: gestational hypertensive disease; Ca: calcium; Pi: phosphate; FGF: fibroblast growth factor.

* p<0.05.

NS: non-significant.

### Comparison of the first three gestational hypertensive disease subgroups with the control group (Table 2)

**Table 2:** Comparison of the gestational hypertensive subgroup and the control group in terms of demographic parameters and laboratory values.

	Control (25)	GHT (5)	Preeclampsia (23)	Eclampsia (2)	p-value
	Mean±standard deviation				
Age (years)	30.72±6.33	25.80±0.77	31.43±6.33	23.50±6.34	NS
Gravity	2.60±1.44	2.20±1.78	2.74±1.88	1.50±0.70	NS
Parity	0.80±0.76	0.60±0.89	1.04±1.10	1.50±0.70	NS
Abortus	0.84±0.98	0.60±0.89	0.87±1.54	0	NS
Birth weight (g)	3126.60±371.24	3048.00±610.98	2995.00±726.20	1482.50±123.74	0.116
Birth weight percentile (%)	45.08±28.48	48.80±35.56	50.21±33.72	3.00±2.82	0.188
Gestational week at delivery	38.13±1.50	37.66±0.84	36.64±1.77	32.00±0	<0.001^a^
Preop Hgb (g/dl)	12.62±1.11	12.56±0.61	12.04±1.04	12.00±0.28	0.261
Postop Hgb (g/dl)	11.12±1.26	11.06±1.41	10.35±1.46	11.70±0.14	0.191
Serum Ca (mg/dl)	8.80±0.48	8.97±0.53	8.46±0.63	7.42±0.36	0.003^b^
Corrected Ca (mg/dl)	9.33±0.38	9.40±0.34	9.14±0.59	9.10±0.58	0.470
Serum Pi (mg/dL)	3.54±0.53	3.86±0.61	3.66±0.82	5.08±1.71	0.283
Systolic (mmHg)	104.40±12.61	142.00±4.47	156.09±17.77	150.00±0.00	<0.001^c^
Diastolic (mmHg)	67.20±7.91	94.00±5.47	99.57±12.60	105.00±7.07	<0.001^d^
FGF23 (pg/mL)	285.09±218.18	330.45±458.00	236.49±287.38	180.10±55.69	0.721

GHT: gestational hypertension; Ca: calcium; Pi: phosphate; FGF: fibroblast growth factor.

a p-value is significant between eclampsia and other groups (p<0.001) and between preeclampsia and control (p=0.035) by Tukey HSD.

b p-value is significant between eclampsia and control (p=0.007) and between eclampsia and GHT (p=0.008) by Tukey HSD.

c p-value is significant between control and other groups (p<0.001) and between preeclampsia and GHT (p=0.015) by Tukey HSD.

d p-value is significant between control and GHT (p<0.001) and between control and preeclampsia (p<0.001) by Tukey HSD.

NS: non-significant.

The gestational week at delivery of the preeclampsia and eclampsia subgroups was earlier than that of the control group, while the gestational week at delivery in the GHT subgroup was similar to the control group. Serum calcium levels in the preeclampsia and eclampsia groups had lower values compared with the control group and the GHT group but were similar to those of the preeclampsia group. However, albumin-corrected calcium levels were similar in all groups. Systolic blood pressure was found to be higher in the GHT, preeclampsia, and eclampsia subgroups compared with the control group (p<0.001). In addition, the preeclampsia subgroup had higher systolic blood pressure than the GHT subgroup (p=0.015). Diastolic blood pressure was higher in the GHT and preeclampsia subgroups than in the control group. However, in the eclampsia group, diastolic blood pressure had higher values than the control group, although there was no statistically significant difference due to the small number of participants. Serum FGF23 levels were similar in the control group and the GHD group. In addition, there was no statistically significant difference in serum FGF23 levels between the control group and the GHT, eclampsia, and preeclampsia subgroups.

### Comparison of the second two gestational hypertensive disease subgroups with the control group (Table 3)

**Table 3:** Comparisons of (1) the gestational hypertension with the absence of the fetal growth restriction group and the control group and (2) the gestational hypertension with the presence of the fetal growth restriction group and the control group in terms of demographic parameters and laboratory values.

	Control (25)	GHD with the absence of FGR (22)	p-value	GHD with the presence of FGR (8)	p-value
	Mean±SD		Mean±SD		
Age (years)	30.72±6.33	30.09±6.14	0.732	29.62±7.34	NS
Gravity	2.60±1.44	2.23±1.47	0.278	3.50±2.39	NS
Parity	0.80±0.76	0.91±1.10	0.900	1.00±0.92	NS
Abortus	0.84±0.98	0.50±0.74	0.232	1.50±2.39	NS
Birth weight (g)	3126.60±371.24	3248.86±553.58	0.321	1951.88±403.83	**<0.001**
Birth weight percentile (%)	45.08±28.48	62.40±24.74	**0.010** *	2.12±1.45	**<0.001**
Gestational week at delivery	38.00±1.53	37.06±1.58	**0.023** *	34.95±2.41	**<0.001**
Preop Hgb (g/dl)	12.62±1.11	12.17±1.11	0.135	12.12±0.30	0.054
Postop Hgb (g/dl)	11.12±1.26	10.54±1.48	0.154	10.65±1.27	0.367
Serum Ca (mg/dL)	8.80±0.48	8.65±0.65	0.329	8.04±0.56	**0.001**
Corrected Ca (mg/dL)	9.33±0.38	9.20±0.57	0.305	9.17±0.47	0.343
Serum Pi (mg/dL)	3.54±0.53	3.60±0.76	0.924	4.30±1.13	0.115
Systolic (mmHg)	104.40±12.61	150.00±20.44	**<0.001**	156.25±13.02	**<0.001**
Diastolic (mmHg)	67.20±7.91	96.96±9.26	**<0.001**	101.25±19.59	**<0.001**
FGF23 (pg/mL)	285.09±218.18	288.26±337.56	0.701	118.95±77.35	**0.044** *

GHD: gestational hypertensive disease; FGR: fetal growth restriction; Ca: calcium; Pi: phosphate; FGF: fibroblast growth factor.

* p<0.05.

NS: non-significant.

Bold indicates statistically significant p-values.

In a comparison of the GHD without FGR group with the control group, the former had a lower gestational week at delivery and a higher BW percentile (p=0.023 and p=0.01, respectively). In addition, higher diastolic and systolic blood pressure values were determined in the GHD without FGR group compared with the control group (p<0.001). FGF23 levels were statistically similar between the GHD without the FGR group and the control group (p=0.073) (Table 3).

In a comparison of the GHD with the FGR group and the control group, the former had a lower gestational week, lower BW, and lower BW percentile (p<0.001). Although serum calcium values were found to be statistically lower in the GHD with the FGR group, the albumin-corrected calcium values were similar when compared with the control group. The GHD with the FGR group had higher systolic and diastolic blood pressure values (p<0.001), while FGF23 levels were statistically significantly lower than those of the control group (p=0.043) (Table 3).

## DISCUSSION

This study showed that serum FGF23 levels detected in normotensive pregnant women and pregnant women with GHT disease were statistically similar, but serum FGF23 levels were found to be low in pregnancies with GHT disease with the presence of FGR. A study on rats reported that maternal malnutrition in the prenatal period resulted in low FGF23 levels. The strong relationship between this FGF23 decrease and FGR was explained in another study^13^. A study conducted to clarify the etiologies of FGR, the direct relationship of FGF23 levels with BW, length, and head circumference determined that the risk of FGR was lower in pregnant women with high FGF23 levels^14^. Similarly, this study showed that maternal serum FGF23 levels were associated with fetal BW, and low FGF23 levels may be a risk factor for FGR.

The relationship between GDM and serum FGF23 levels has been investigated in previous studies. Serum FGF23 levels were found to be high in pregnancies complicated with GDM^15^; however, FGF23 levels were examined in pregnant women at an early gestational week. In addition, neither estimated fetal weight nor BW were noted in the study. Another reason that FGF23 levels have been found to be higher in pregnancies complicated with GDM may be due to the high estimated fetal weight or BW. The result of this study is supported by the positive correlation between BW and BW percentile and FGF23 levels.

A recent study found that high α-Klotho values in human serum value can be considered a marker for preeclampsia^16^. Miranda et al. also found that maternal serum α-Klotho concentrations were higher than in non-pregnant women, but this increase was not significant in pregnant women with FGR fetuses regardless of preeclampsia^17^. In this study, we investigated FGF23, which is the obligate receptor of α-Klotho, but failed to detect significant differences in the serum FGF23 levels of the GHD group (including thepreeclampsia, eclampsia, and GHT subgroups) and the control group. However, there was a correlation between FGF23 levels and BW.

It is known that elevated FGF23 concentrations may occur due to iron deficiency and that iron replacement reverses this elevation. In addition, it has been shown that high FGF23 levels cause osteomalacia and rickets through phosphate and calcium metabolism and that iron replacement in pregnancy reverses the decrease in bone mineral density by decreasing FGF23 levels^18^. Although maternal serum ferritin levels were not identified in this study, maternal Hgb concentrations were found to be similar in all the groups. In addition, it should be noted that oral iron therapy is administered to all pregnant women in our country.

Previous studies have shown that, especially in the last trimester, there is a greater increase in blood pressure in pregnancies complicated with preeclampsia than in pregnancies complicated with GHT^19^. Davis et al. found that pregnant women with GHT who progressed to preeclampsia had higher blood pressure values than GHT patients who did not progress to preeclampsia^20^.

In our study, similar to the literature, we found that systolic blood pressure was higher in the preeclampsia group than in the GHT group, but diastolic blood pressure was similar in the two groups. This may be due to the small number of participants in the GHT group. Studies with a larger number of participants are needed to determine the accuracy of this finding.

Another interesting finding of our study was that the total serum calcium level was lower in the eclampsia subgroup than in the control group and the GHT subgroup. However, we found that albumin-corrected calcium levels were similar in all groups. The relationship between low maternal calcium levels and pregnancy-induced hypertensive diseases has been explained, but the protectiveness of calcium replacement in pregnancy for GHD has not yet been clearly demonstrated^21^. In a recent study, serum calcium levels were found to be lower in preeclamptic pregnant women compared with healthy pregnant women^22^. On the contrary, in our study, neither serum total calcium levels nor albumin-corrected calcium levels of the preeclampsia group were similar to those of the control group. A decrease in total serum calcium levels was demonstrated only in the eclamptic pregnant women.

Although the strongest limitation of our study was the inability to evaluate FGF23 levels together with serum and/or placental α-Klotho values, the relationship between FGF23 levels and FGR was demonstrated. Additional limitations include not knowing whether the participants took oral iron and/or multivitamin supplements and the small number of participants, especially in the eclampsia group. In addition, the index study group is small; therefore, further research is needed to validate this approach.

In conclusion, in this study, serum FGF23 levels were similar in pregnancies complicated by GHD and normotensive pregnancies. However, serum FGF23 levels were found to be lower in pregnancies complicated with GHD with the presence of FGR.

## ETHICS

The study was performed in accordance with the ethical standards for human research established by the Declaration of Helsinki and Good Clinical Practice guidelines and was approved by the local Ethics Committee of Süleyman Demirel University School of Medicine.

## INFORMED CONSENT

All patients provided written informed consent for the application of this technique.
